# A Study of the Adsorption Properties of Individual Atoms on the Graphene Surface: Density Functional Theory Calculations Assisted by Machine Learning Techniques

**DOI:** 10.3390/ma17061428

**Published:** 2024-03-20

**Authors:** Jingtao Huang, Mo Chen, Jingteng Xue, Mingwei Li, Yuan Cheng, Zhonghong Lai, Jin Hu, Fei Zhou, Nan Qu, Yong Liu, Jingchuan Zhu

**Affiliations:** 1 School of Materials Science and Engineering, Harbin Institute of Technology, Harbin 150001, China; 20b909032@stu.hit.edu.cn (J.H.); lixlion@163.com (M.C.); xuejingteng.cn@gmail.com (J.X.); hujin@hit.edu.cn (J.H.); 2 National Key Laboratory for Precision Hot Processing of Metals, Harbin Institute of Technology, Harbin 150001, China; limingwei@hit.edu.cn; 3 National Key Laboratory of Science and Technology on Advanced Composites in Special Environments, Harbin Institute of Technology, Harbin 150001, China; cy6810@hit.edu.cn; 4 Center for Analysis, Measurement and Computing, Harbin Institute of Technology, Harbin 150001, China; zhhlai@hit.edu.cn; 5 State Key Laboratory for Environment-Friendly Energy Materials, School of Materials Science and Engineering, Southwest University of Science and Technology, Mianyang 621010, China; fei_flyfly@163.com

**Keywords:** single atoms, graphene, surface adsorption, first-principles calculations, machine learning

## Abstract

In this research, the adsorption performance of individual atoms on the surface of monolayer graphene surface was systematically investigated using machine learning methods to accelerate density functional theory. The adsorption behaviors of over thirty different atoms on the graphene surface were computationally analyzed. The adsorption energy and distance were extracted as the research targets, and the basic information of atoms (such as atomic radius, ionic radius, etc.) were used as the feature values to establish the dataset. Through feature engineering selection, the corresponding input feature values for the input-output relationship were determined. By comparing different models on the dataset using five-fold cross-validation, the mathematical model that best fits the dataset was identified. The optimal model was further fine-tuned by adjusting of the best mathematical ML model. Subsequently, we verified the accuracy of the established machine learning model. Finally, the precision of the machine learning model forecasts was verified by the method of comparing and contrasting machine learning results with density functional theory. The results suggest that elements such as Zr, Ti, Sc, and Si possess some potential in controlling the interfacial reaction of graphene/aluminum composites. By using machine learning to accelerate first-principles calculations, we have further expanded our choice of research methods and accelerated the pace of studying element–graphene interactions.

## 1. Introduction

Two-dimensional materials have attracted a great deal of attention in the scientific community, and in recent years, researchers have explored the unique electronic properties of single- and low-layer samples of these materials to develop new applications in the fields of electronics, photonics, chemical sensing, catalysis, transport properties, and energy storage. In addition, the rapid development of graphene and the preparation of ultra-thin layers have triggered the exploration of other two-dimensional materials. The basic chemical properties of graphene are similar to those of graphite, and its most basic chemical bond is also a carbon-carbon double bond, but graphene has fewer layers of hexagonal honeycomb junctions, which gives graphene different chemical properties from graphite, i.e., graphene can adsorb and desorb a variety of atoms and molecules. Graphene, with its excellent optical, electrical, and mechanical properties, has important application prospects in materials science, micro- and nanofabrication, energy, biomedicine, and drug delivery and is considered to be a revolutionary material for the future. In the last few years, graphene/aluminum composites have received extensive attention from a wide range of scholars. However, graphene is prone to severe interfacial reactions with conventional aluminum alloys, which in turn reduces the mechanical properties of the composites. The analysis and prediction of material properties using machine learning (ML) methods has become a hot research topic in the interdisciplinary field of material science and computer science [1,2]. With the rapid development of computer science and technology, ML algorithms have been widely applied. ML tools have broad applications in various fields, such as natural language processing, image recognition, object detection, pattern recognition, and robotics [3,4,5]. In recent years, significant research achievements have also been continuously made in the implementation of ML techniques in material analysis and design [6,7]. ML has obvious advantages in predicting materials and rapidly discovering new materials; it can shorten the research and development cycle and improve the design efficiency of new materials [8,9,10,11]. On the other hand, potential relationship information hidden in material data can be automatically excavated, greatly promoting theoretical research in the study of materials. Therefore, we will combine first principles and ML algorithms to predict graphene adsorption atomic behavior, aiming to find suitable elemental species to reduce the interfacial reaction between graphene and aluminum alloys.

Machine learning represents one of the most exciting fields in information technology today. As computers and networks continue to advance, machine learning is becoming increasingly valuable and holds the potential to transform our everyday lives and work. Among the current trends in the realm of materials science, the selection of high-performance novel materials and the modeling of quantitative structure-property relationships are being focused on. In recent years, the utilization of ML in materials exploration and development has gained significant recognition and has made notable advancements in terms of temporal effectiveness and forecast precision. Balachandran et al. [12] proposed an adaptive learning method to screen materials with desired structural features, which played a significant role in expediting the innovation and advancement of novel materials. Takahashi et al. [13] used a dataset obtained through first-principles calculations and successfully predicted the crystal structure types of materials using the support vector machine algorithm. Rupp et al. [14] accurately predicted the molecular atomization energy using a Coulomb matrix-based molecular material quantification descriptor, with an average error of less than 10 Kcal/mol. Schut et al. [8] proposed a crystal structure descriptor based on partial radial basis distributions and used it to provide an accurate prediction of the electronic density of states at the Fermi level. In addition, researchers have used support vector machine algorithms to classify metal/insulator materials [15]. Isayev et al. [16] innovatively proposed the attribute-labeled material fragment approach based on graph theory to describe the crystal structure of materials. Hence, dimensionality reduction methods commonly used in ML, such as principal component analysis and singular value decomposition, are employed and can be utilized to map high-dimensional representations of material structures or property information to low-dimensional feature spaces for subsequent research on material structure-property prediction classification, and other related work.

In recent years, first-principles computational methods have gained popularity in materials design as a means to expedite the material design process. The utilization of density functional theory significantly enhances the precision of calculations and ensures the reliability of obtained results. The time cost of first-principles calculations, in comparison to machine learning, is relatively high and can hinder the progress of new materials development. There has been a flurry of research on graphene [17,18,19,20,21], making it a hot topic in the field of materials. Although the use of first-principles calculations [22,23] alone can continuously improve the theoretical understanding of graphene and facilitate the design of new structures, the development cycle remains excessively long. Therefore, it is of great significance to explore new research methods to assist in accelerating the development of graphene materials. To address these issues, this paper proposes an ML-based approach to accelerate first-principles calculations for studying the adsorption performance of graphene towards individual atoms. The focus is on predicting the adsorption energy and adsorption distance of graphene using ML methods, which can help subsequent researchers quickly identify materials with ideal properties. Additionally, we leverage the trained ML model to identify the important features that influence adsorption distance and adsorption energy. The best-performing model was subsequently further optimized using a genetic algorithm, and the hyper-parameters were adjusted based on the root mean squared error to obtain the final model. This final model was then utilized for predicting other remaining elements in the periodic table. The results suggest that elements such as Zr, Ti, Sc, and Si possess some potential in controlling the interfacial reaction of graphene/aluminum composites. The paper is structured into the following sections: Section 2—Methods, Section 3—Results and Discussion, and Section 4—Conclusions.

## 2. Methods

### 2.1. Crystal Structure and Calculations Method

Various ML algorithms were designed to predict the results (the absorption energy and absorption distance of adsorption between different atoms and graphene); the calculation is performed via first-principles calculation by utilizing the Cambridge Sequential Total Energy Package (CASTEP) simulation package [24]. The Perdew–Burke–Ernzerhof (PBE) functional [25,26], which is a generalized gradient approximation (GGA) method, is used to study the exchange–correlation effect. LDA and GGA are two commonly employed density functionals for density-functional theory [27,28,29] calculations in the field of computational materials science. Density Functional Theory is a method for studying the electronic structure of multi-electron systems. Density functional theory has a wide range of applications in both physics and chemistry, especially for studying the properties of molecules and condensed states, and is one of the most commonly used methods in the fields of condensed matter physics, computational materials science, and computational chemistry. It is based on the principles of physics and the theory of quantum mechanics and calculates the behavior of electrons in molecular and material structures by solving the Schrodinger equation. LDA approximates the exchange–correlation function based on the local electron density, whereas GGA accounts for the density gradient. The choice of function depends on the system under investigation and the properties of interest. It is important to note that LDA may not accurately describe certain system properties, particularly the band gap of semiconductors and insulators, which tends to be underestimated. Consequently, the selection of either LDA or GGA should be based on the specific requirements of the material system and the properties of interest. In our case, since graphene is a zero-bandgap semiconductor, GGA is the more suitable choice for determining calculation parameters in this paper. The plane wave uses a set cut-off energy of 450 eV, and the Brillouin zone k-point is 4×4×1. A higher iterative convergence accuracy ($2\times 10^{-6}$ eV/atom) has been used; each atom is subjected to a force that is kept below 0.05 eV/Å, while the internal stress is ensured to not exceed 0.1 GPa.

### 2.2. Machine Learning Databases and Models

**Figure 1:**
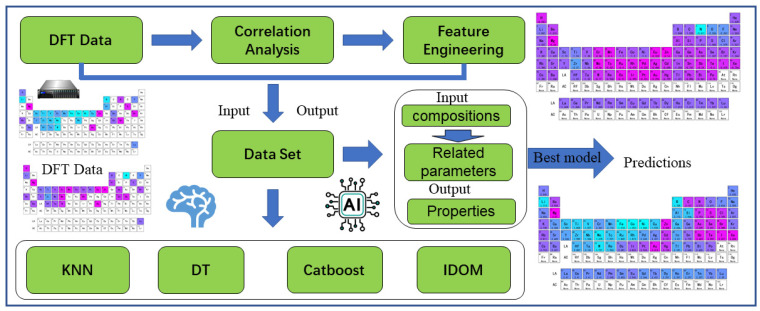
Schematic diagram of the machine learning process.

To overcome the challenges that DFT calculations pose to computer hardware, we have designed various ML algorithms to predict the DFT calculation results. As shown in Figure 1, the ML solution for accelerating the prediction of graphene adsorption properties includes three steps: (1) establishment of the adsorption properties database, (2) establishment and selection of ML models, and (3) prediction of adsorption properties for elements in the periodic table. The machine learning process accelerates DFT computation through three parts: data generation, model construction, and prediction. Cross-validation divides the dataset into smaller subsets and loops them as validation sets, which can improve the reliability of small sample machine learning results. The genetic algorithm obtains optimal connection weights through a cycle of selection, crossover, and mutation. Compared with traditional DFT calculations, machine learning methods have shown advantages in terms of time cost. Our machine learning method aims to establish a regression relationship between the adsorption performance of different atoms and graphene based on a limited number of DFT calculation results. The ML process speeds up DFT calculations through data generation, model construction, and prediction. The genetic algorithm obtains the optimal connection weights through selection, crossover, and mutation cycles. By comparing multiple ML algorithms, we have adopted the best-fitting algorithm to establish four ML models, respectively, predicting adsorption energy and adsorption distance. Four ML models, namely K-nearest neighbor (KNN), decision tree (DT), Catboost, and Input Layer Drop-Out Multilayer(IDOM), are constructed using an optimized algorithm for predicting both adsorption energy and adsorption distance, respectively. CatBoost is a comprehensive decision tree approach that caters to classification features and provides model interpretation. KNN is an instance-based learning method that computationally predicts the distance between a new data point and each data point in the training dataset. In the machine learning model training process, we divided the dataset into two parts: a training set (80%) and a validation set (20%). In the machine learning model accuracy validation process, we use the validation set calculated by DFT to compare with the machine learning model prediction results.

## 3. Results and Discussion

**Figure 2:**
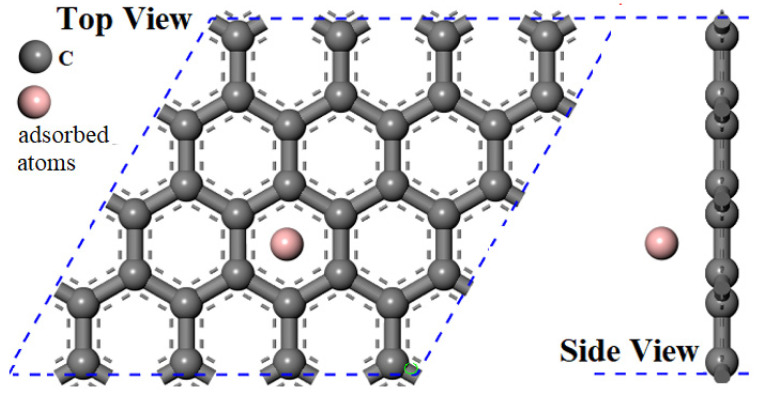
Single atom adsorbed graphene surface model, the gray ones are carbon atoms, and the purple ones are adsorption atoms.

As depicted in Figure 2, the carbon atoms are denoted by gray, and the adsorbed atoms are represented by purple. A total of 32 C atoms and one adsorbed atom are contained, all of which are adsorbed at the locations of the six-membered ring pores of graphene. As described above, all surface geometry is modeled as a periodic flat plate system, with a 20 Å vacuum between the surfaces sufficient to prevent interaction between periodic images. The adsorption energy is calculated using the following equation [30]:

(1) $$E_{ads}=E_{\mathrm{total}\;}-E_{v}-E_{\mathrm{atom}\;}$$

The total energy of graphene following the adsorption of single atoms is denoted by $E_{\mathrm{total}}$, while the total energy of graphene is represented by $E_{v}$, and the energy of the adsorbed atom is represented by $E_{\mathrm{atom}}$. The results for the distance and energy of adsorption can be found in Table S1.

Using data obtained from DFT calculations, we compiled a set of more than ten kinds of characteristic parameters to assess the adsorption distance and adsorption energy. These parameters include the Atomic number, Atomic radius, Ionic radius, Covalent radius, Atomic volume, Relative atomic mass, Period, Electron configuration (s), Electron configuration (p), Electron configuration (d), Electron configuration (f), Electron affinity, 1st ionization energy, 2nd ionization energy, 3rd ionization energy, and Group.

### 3.1. Correlation Analysis and Selection of Eigenvalues

Correlation analysis refers to the analysis of two or more elements of a variable that are correlated in order to measure the closeness of the correlation between two elements of the variable. There needs to be a certain link or probability between the correlated elements for correlation analysis to take place. The data regarding the distance and energy of adsorption between graphene and thirty-four elements, obtained through DFT calculations, were acquired as shown in Tables S1 and S2. Furthermore, more than ten kinds of features were compiled to assess the adsorption energy and adsorption distance between graphene and various elemental atoms, encompassing atomic number, atomic volume, et al. These data are collectively displayed in Table S1, which served as the raw data used.

Further, we make a selection of descriptors. The adsorption distance descriptors and adsorption energy descriptors were screened from the above 16 characteristic quantities. The Pearson correlation coefficient is an indicator of the degree of correlation between response variables. Pearson correlation coefficient was calculated to find out the factors that had the greatest influence on adsorption distance and adsorption energy, and then their respective descriptors were determined. The descriptors for the distance and energy of adsorption were separately selected from more than ten kinds of features mentioned above. The correlation strength among the response variables is measured by the correlation coefficient, serving as an indicator. Factors that have the most significant impact on the adsorption distance and adsorption energy were identified through the calculation of the correlation coefficient, respectively. Their respective descriptors were determined through this process. The formula used to calculate the correlation coefficient is expressed as follows [31,32,33]:

(2) $$\rho_{X,Y}=\frac{\sum \left(X_{i}-\bar{X}\right)\left(Y_{i}-\bar{Y}\right)}{\sqrt{\sum {\left(X_{i}-\bar{X}\right)}^{2}\sum {\left(Y_{i}-\bar{Y}\right)}^{2}}}$$

$\rho_{XY}$ represents the Pearson correlation coefficient. $X_{i}$ and $Y_{i}$ refer to the eigenvalues and target values, while $\bar{X}$ and $\bar{Y}$ represent the averages of X and Y, respectively. The Pearson correlation coefficient ranges from −1 to 1 in magnitude, where 0 indicates no correlation between the variables.

**Figure 3:**
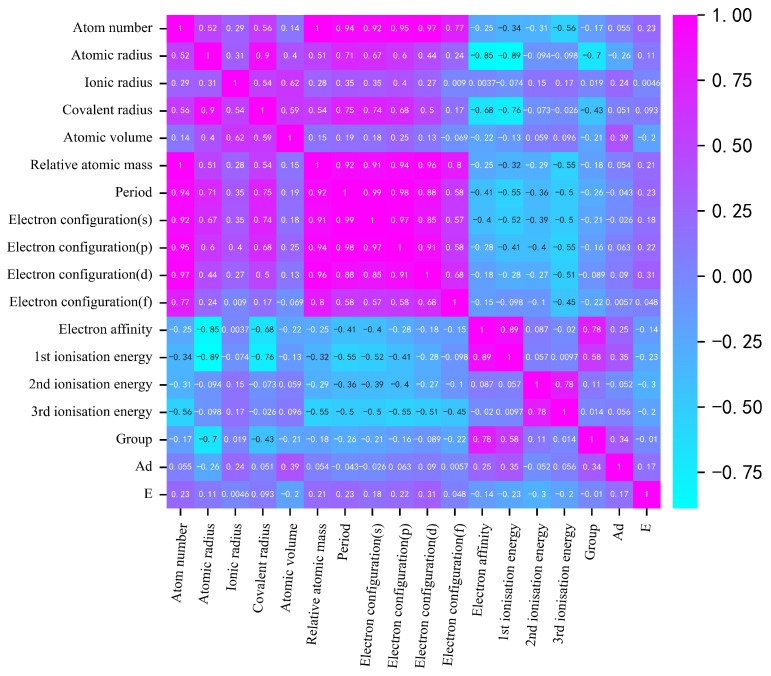
Correlation analysis results of eigenvalue inputs and outputs.

As demonstrated in Figure 3, the electron configuration (d) and atomic volume exhibit the most significant impact on the distance and energy of adsorption; the Pearson correlation coefficients were found to be 0.31 and 0.39, respectively. The Pearson correlation coefficients of s, p, d orbital and adsorption energy reached 0.18, 0.22, and 0.33, respectively. The size of the atomic volume is positively correlated with the adsorption distance, meaning that as the atomic volume increases, there is a tendency for the adsorption distance to increase. Consideration of various factors allows for the identification of descriptors that determine the adsorption energy magnitude, such as the covalent radius, Atomic volume, Electron affinity, 1st ionization energy, and Group. The descriptors determining the adsorption distance are identified as the Atomic radius, Covalent radius, Electron affinity, 1st ionization energy, 2nd ionization energy.

The descriptors of the distance and energy of adsorption, along with their corresponding target data, are used to construct the training data for the ML model based on the correlation analysis conducted above. The input data for the ML dataset are served by the descriptors, while the target data comprises the adsorption energy and adsorption distance. Table S2 presents the database of adsorption performance, which is based on ML. In order to ensure that all variables fall within the same range, both input and output variables are normalized between 0 and 1 during the training process using the following equation [34]:

(3) $$X_{i}^{\prime }=\frac{X_{i}-X_{\min}}{X_{\max}-X_{\min}}$$

where $X_{i}$ represents the data individual, $X_{\max}$ is the maximum value within that specific category of data that needs to be determined and analyzed accurately, and $X_{\min}$ is the minimum value.

### 3.2. Machine Learning Model Building and Selection

In order to accurately evaluate the performance of different ML models when applied to novel data and optimize data utilization, cross-validation methods are utilized. The utilization of cross-validation, a statistical technique that assesses a model’s ability to generalize by dividing the dataset into distinct partitions, enables the achievement of this goal. In this section, a commonly used 5-fold cross-validation approach was employed. With this approach, the original data was initially divided into five subsets in a random manner. Subsequently, the model was trained and validated five times. During each iteration, the model was trained on four subsets, forming the training set, and then tested on the remaining subset, referred to as the validation set. This process was repeated five times, and the outcomes were averaged to obtain more accurate estimations of the model’s performance. The mentioned algorithms were implemented in Python, utilizing scientific computing packages such as pandas and numpy. In our study, three evaluation metrics were introduced to assess the effectiveness of various models: mean square error (MSE), root mean square error (RMSE), mean absolute percentage error (MAPE), and coefficient of determination ($R^{2}$). The calculations for MSE, RMSE, MAPE and $R^{2}$ are outlined as follows [35]:

(4) $$\mathrm{MSE}=\frac{1}{N}\sum_{i=1}^{N}{\left(y_{i}-\hat{y}\right)}^{2}$$

(5) $$RMSE=\sqrt{\frac{1}{N}\sum_{i=1}^{N}{\left(y_{i}-\hat{y}\right)}^{2}}$$

(6) $$\mathrm{MAPE}=\frac{1}{N}\sum_{i=1}^{N}\left|\frac{y_{i}-{\hat{y}}_{i}}{y_{i}}\right|\times 100\%$$

(7) $$R^{2}=1-\frac{\sum_{i=1}^{N}{\left(y_{i}-\hat{y}\right)}^{2}}{\sum_{i=1}^{N}{\left(y_{i}-\bar{y}\right)}^{2}}$$

where $\hat{y_{i}}$ is the ML algorithm predicted value and $y_{i}$ is the DFT calculated value, $\bar{y_{i}}$ is the mean of the calculated DFT results, where N is the number of samples.

**Figure 4:**
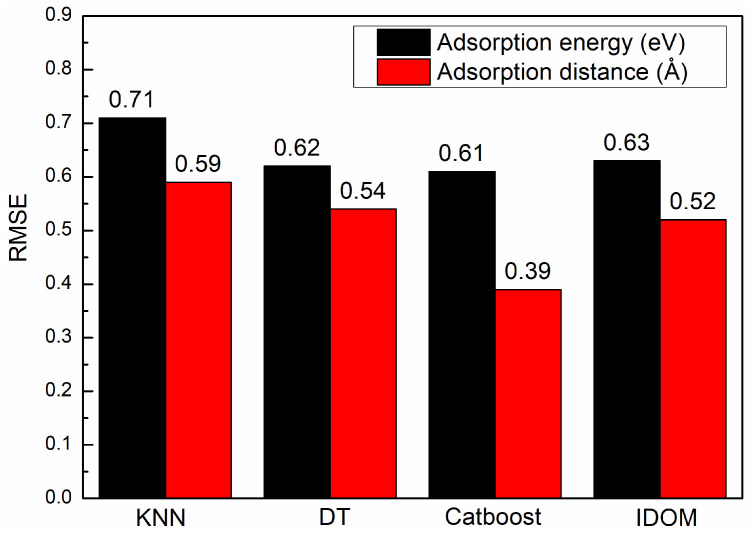
Root mean square error corresponding to different learning models.

**Figure 5:**
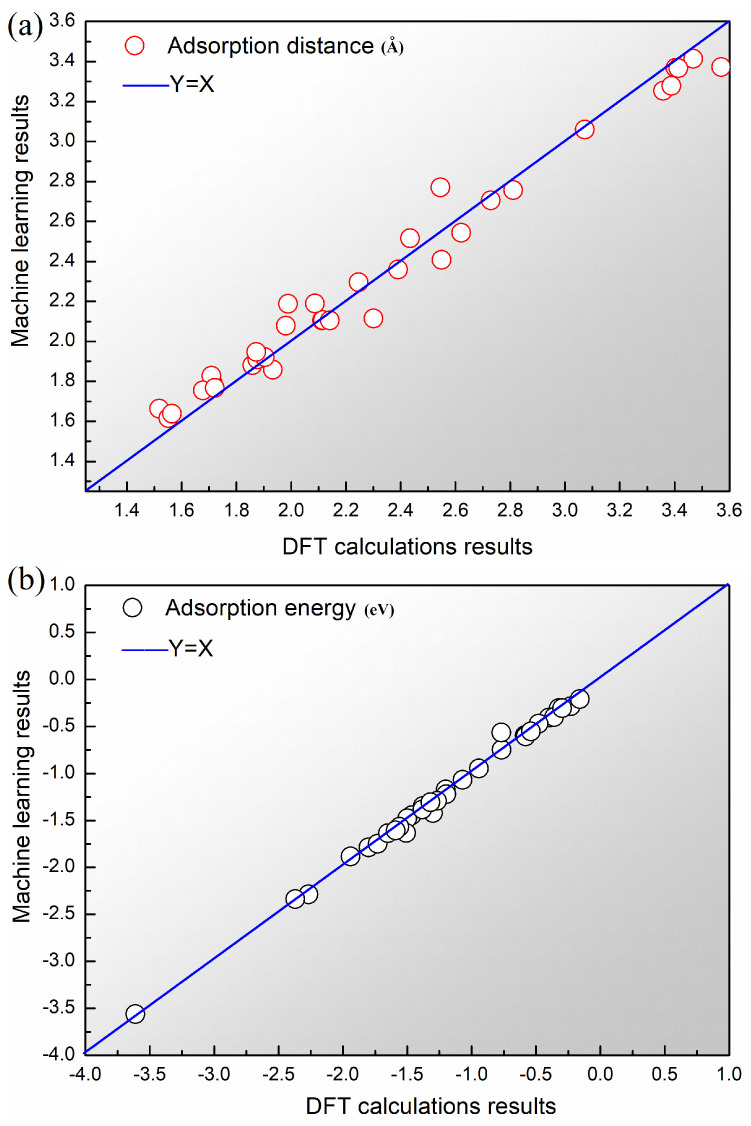
Scatter plots representing the regression results of the complete dataset using the Catboost model for (**a**) adsorption distance and (**b**) adsorption energy.

It is indicated by the cross-validation results of various algorithms that the other algorithms are significantly outperformed by Catboost, as shown in Figure 4. The worse performance is observed in the KNN algorithm. The cause of these results can be attributed to the requirement of a significant number of training parameters in the neural network, making it unsuitable for this small sample data problem. A notable advantage over the traditional DT algorithm is demonstrated by Catboost among the tree-based algorithms. In the predictions of adsorption distance and adsorption energy, it achieves an RMSE of 0.61 and 0.39, respectively. Regression analysis confirms the substantial correlation between the ML predicted values and the DFT calculated values, and a well-fitted model is indicated by the even distribution of data samples around the reference line (Y=X), as shown in Figure 5. Errors for Ed and G are 0.96% and 0.28%, respectively, meeting the target accuracy requirements. Therefore, the Catboost algorithm was selected as the model for subsequent ML tasks.

### 3.3. Prediction and Verification of Results

**Figure 6:**
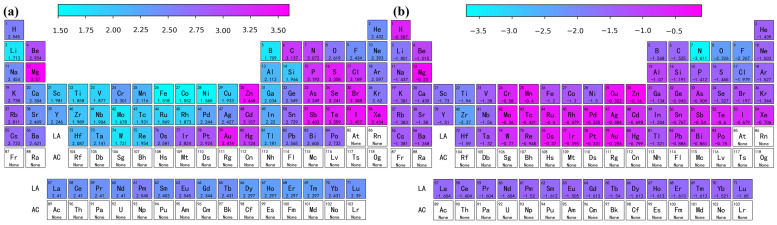
Full periodic table display of (**a**) adsorption energy and (**b**) adsorption distance.

A trained Catboost algorithm ML model was used to predict the energy and distance of adsorption for the remaining elements of the periodic table when adsorbed on the surface of graphene as shown in Table 1. However, the absence of partial descriptors for certain rare elements, such as lanthanides and actinides, was not taken into account in this study. The best performing Catboost model identified in step 3.2 was employed to estimate the adsorption energy and distance for the remaining elements. The predicted energy and distance of adsorption for different elemental atoms on the surface of graphene are illustrated in Figure 6. It is very important to select appropriate elements to inhibit the interfacial reaction of graphene/aluminum composites. From Figure 6a, we can see that elements such as Li, B, Zr, Ti, Sc, Si, and Ta have relatively low adsorption distances when adsorbed on a graphene surface. From Figure 6b, we can see that elements such as N, O, Zr, Sc, Ti, and Si have relatively low adsorption energy when adsorbed on the graphene surface. The above results suggest that elements such as Zr, Ti, Sc, and Si possess some potential in controlling the interfacial reaction of graphene/aluminum composites.

**Figure 7:**
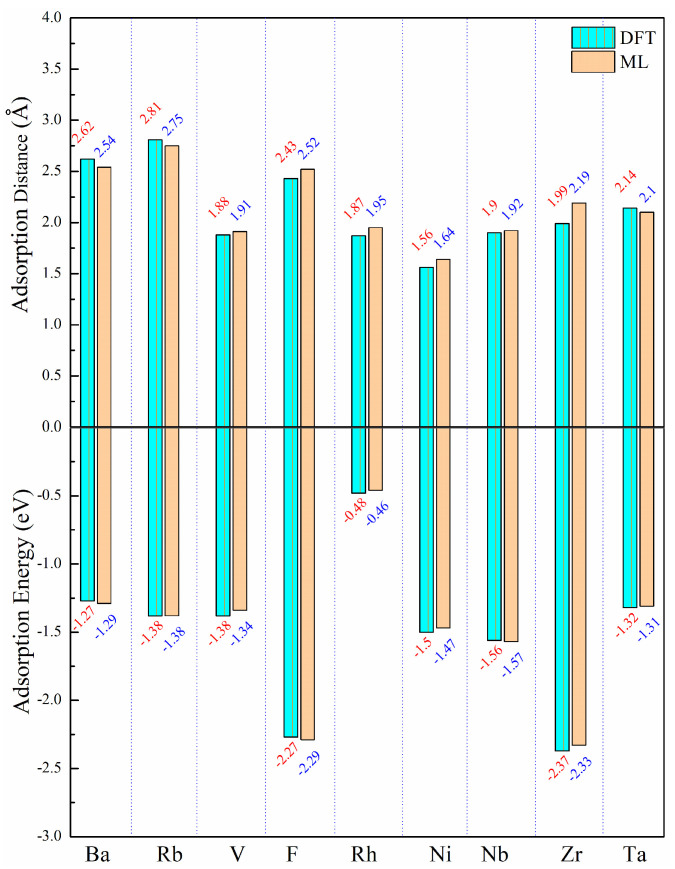
Comparison of adsorption energy and adsorption distance predicted by the Catboost model with DFT results.

The computed results and ML predictions were compared with the research findings by Pasti et al. [9], as presented in Table S3. It is apparent that both the computed results and prediction results closely match the results of Pasti et al. [9], and the reliability of our calculations and the accuracy of our ML predictions are affirmed. The demonstration of the remarkable efficiency of the ML approach for predicting adsorption energy and distance highlights its superiority over DFT-based calculations, considering that it is tens of thousands of times faster. This accelerated approach to material calculations using ML provides a solution to the limitations of inefficient DFT calculations and significantly reduces computational costs. To ensure the accuracy and reliability of the ML prediction results, a comparison was made between the ML predictions and the DFT calculation results in the dataset, as shown in Figure 7. The results presented in Figure 7 unmistakably establish the validity and credibility of the ML predictions, as they exhibit minimal relative error compared to the results obtained through first principle calculations.

## 4. Conclusions

In this paper, we investigate the adsorption behavior of single atoms adsorbed on the surface of graphene using machine learning-accelerated first-principles calculations. The behavior of individual atoms adsorbed on the graphene surface has been investigated through accelerated first-principles calculations enhanced by machine learning techniques. We generated a database by computing the energy and distance of adsorption using first-principles calculations. In order to improve ML performance, we utilized characteristic parameters like atomic and ionic radii to create a dataset. Multiple ML models, such as KNN, DT, Catboost, and IDOM, were utilized to construct mathematical models. Based on decision coefficients and root mean square error, it was indicated that the dataset was best suited for the Catboost model. The Catboost model was further refined with the aim of enhancing the coefficient of determination and minimizing the root mean square error. The adsorption behavior of atoms across the entire periodic table was anticipated using the subsequently employed refined Catboost model. In order to verify the accuracy of these predictions, we compare the ML predictions with first-principles calculations, which are shown to have a very low error. The development time was substantially reduced by integrating ML techniques to expedite the first-principles approach, thereby facilitating expedited research in the field of elemental modifications of graphene. We pick out a series of elements that are appropriate to inhibit the interface reaction; elements such as Li, B, Zr, Ti, Sc, Si, and Ta have relatively low adsorption distances when adsorbed on graphene surface, and elements such as N, O, Zr, Sc, Ti, and Si have relatively low adsorption energy when adsorbed on the graphene surface, elements such as Zr, Ti, Sc, and Si possess some potential in controlling the interfacial reaction of graphene/aluminum composites. This study offers a comprehensive understanding of the adsorption properties of single atoms from the entire periodic table on the surface of graphene, enabling experimental modifications of graphene.

## Figures and Tables

**Table 1:** MSE (${\mathrm{eV}}^{2}$ for adsorption energy and ${Å}^{2}$ for adsorption distance), $R^{2}$, RMSE (eV for adsorption energy and Å for adsorption distance), MAPE of the final model.

	MSE	$R^{2}$	RMSE	MAPE
Adsorption distance	0.0096	0.9752	0.0982	3.6181
Adsorption energy	0.0028	0.9946	0.0525	4.5995

## Data Availability

Data are contained within the article and Supplementary Materials.

## Supplementary Materials

The following supporting information can be downloaded at: https://www.mdpi.com/article/10.3390/ma17061428/s1, Figure S1. Variation of mean square error with the number of eigenvalues in feature elimination; Table S1. Final selection of feature values to be used as a machine learning dataset for adsorption distance. Table S2. Final selection of feature values to be used as machine learning dataset. Table S3. Comparison of adsorption energy results with first-principle calculations by other scholars.
