# Supplementary material for: A Study of the Adsorption Properties of Individual Atoms on the Graphene Surface: Density Functional Theory Calculations Assisted by Machine Learning Techniques
Source: Materials (Basel). 2024 Mar 20;17(6):1428. doi: 10.3390/ma17061428 (PMC10971905; doi:10.3390/ma17061428)
Supplement: Supplementary file 1 [file materials-17-01428-s001.zip › materials-2656561-supplementary.pdf]

Supplemental Information

A study of the adsorption properties of individual atoms on the graphene surface: machine learning accelerated density functional theory

Jingtao Huang [1], Mo Chen [1], Jingteng Xue [1], Mingwei Li [2], Yuan Cheng [3], Zhonghong Lai [4], Jin Hu [1], Fei Zhou [5], Nan Qu [1,*], Yong Liu [1,2,*,†] and Jingchuan Zhu [1,*,†]

[1] School of Materials Science and Engineering, Harbin Institute of Technology, Harbin 150001, China; 20b909032@stu.hit.edu.cn (J.H.); lixlion@163.com (M.C.); xuejingteng.cn@gmail.com (J.X.); hujin@hit.edu.cn (J.H.)

[2] National Key Laboratory for Precision Hot Processing of Metals, Harbin Institute of Technology, Harbin 150001, China; limingwei@hit.edu.cn

[3] National Key Laboratory of Science and Technology on Advanced Composites in Special Environments, Harbin Institute of Technology, Harbin 150001, China; cy6810@hit.edu.cn

[4] Center for Analysis, Measurement and Computing, Harbin Institute of Technology, Harbin 150001, China; zhhlai@hit.edu.cn

[5] State Key Laboratory for Environment-Friendly EnergyMaterials, School ofMaterials Science and Engineering, Southwest University of Science and Technology, Mianyang 621010, China; fei_flyfly@163.com

***** Correspondence: qunan@hit.edu.cn (N.Q.); lyonghit@hit.edu.cn (Y.L.); fgms@hit.edu.cn (J.Z.)

† These authors contributed equally to this work.

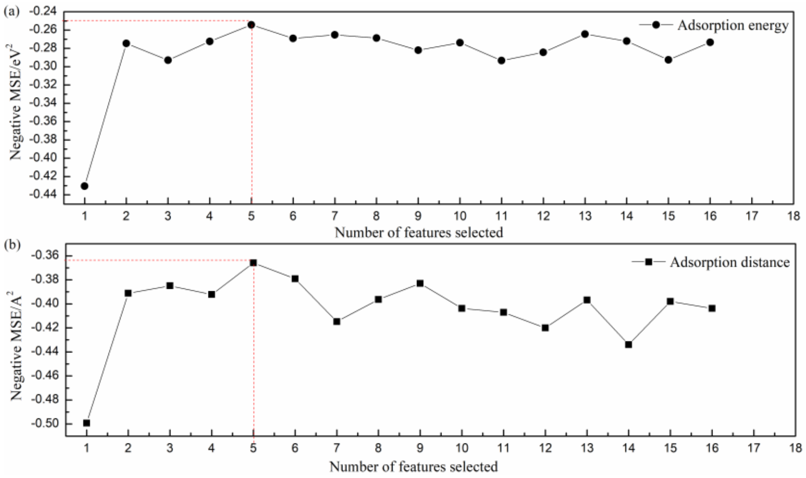

Figure S1. Variation of mean square error with the number of eigenvalues in feature elimination.

Table S1. Final selection of feature values to be used as a machine learning dataset for adsorption distance.

Atomic Name	Atomic radius	Covalent radius	Electron affinity	1st ionization energy	2nd ionization energy	A_d
H	0.79	0.32	2.2	1312	0	2.545
Li	2.05	1.23	0.98	520.2	7298.1	1.713
B	1.17	0.82	1.92	800.6	2427.1	1.709
N	0.75	0.75	3.04	1402.3	2856	3.072
F	0.57	0.72	3.98	1681	3374.2	2.434
Mg	1.72	1.36	1.31	737.7	1450.7	3.57
Al	1.82	1.18	1.61	577.5	1816.7	2.113
S	1.09	1.02	2.58	999.6	2252	3.358
Sc	2.09	1.44	1.36	633.1	1235	1.981
Ti	2	1.32	1.54	658.8	1309.8	1.858
V	1.92	1.22	1.63	650.9	1414	1.877
Cr	1.85	1.18	1.66	652.9	1590.6	2.301
Mn	1.79	1.17	1.55	717.3	1509	2.116
Fe	1.72	1.17	1.83	762.5	1561.9	1.518
Co	1.67	1.16	1.88	760.4	1648	1.552
Ni	1.62	1.15	1.91	737.1	1753	1.565
Cu	1.57	1.17	1.9	745.5	1957.9	1.933
Zn	1.53	1.25	1.65	906.4	1733.3	3.468
Ge	1.52	1.22	2.01	762	1537.5	2.549
Br	1.12	1.14	2.96	1139.9	2103	3.388
Rb	2.98	2.16	0.82	403	2633	2.811
Y	2.27	1.62	1.22	600	1180	2.246
Zr	2.16	1.45	1.33	640.1	1270	1.989
Nb	2.08	1.34	1.6	652.1	1380	1.904
Mo	2.01	1.3	2.16	684.3	1560	1.678
Rh	1.83	1.25	2.28	719.675	1744.45	1.873
Sn	1.72	1.41	1.96	708.6	1411.8	2.729
Sb	1.53	1.4	2.05	834	1594.9	3.403
Ba	2.78	1.98	0.89	502.9	965.2	2.621
Lu	2.25	1.56	1	523.5	1340	2.39
Hf	2.16	1.44	1.3	658.5	1440	2.087
Ta	2.09	1.34	1.5	761	1500	2.141
W	2.02	1.3	1.7	758.764	1553.4	1.721
Au	1.79	1.34	2.4	890.1	1980	3.414

Table S2. Final selection of feature values to be used as machine learning dataset for adsorption energy.

Atomic Name	Covalent radius	Atomic volume	Electron affinity	1st ionization energy	Group	E
H	0.32	14.4	2.2	1312	1	-0.587
Li	1.23	13.1	0.98	520.2	1	-1.801
B	0.82	4.6	1.92	800.6	13	-1.568
N	0.75	17.3	3.04	1402.3	15	-3.611
F	0.72	17.1	3.98	1681	17	-2.267
Mg	1.36	13.97	1.31	737.7	2	-0.23
Al	1.18	10	1.61	577.5	13	-1.07
S	1.02	15.5	2.58	999.6	16	-1.465
Sc	1.44	15	1.36	633.1	3	-1.73
Ti	1.32	10.64	1.54	658.8	4	-1.94
V	1.22	8.78	1.63	650.9	5	-1.38
Cr	1.18	7.23	1.66	652.9	6	-0.58
Mn	1.17	1.39	1.55	717.3	7	-0.4
Fe	1.17	7.1	1.83	762.5	8	-1.2
Co	1.16	6.7	1.88	760.4	9	-1.3
Ni	1.15	6.59	1.91	737.1	10	-1.5
Cu	1.17	7.1	1.9	745.5	11	-0.322
Zn	1.25	9.2	1.65	906.4	12	-0.16
Ge	1.22	13.6	2.01	762	14	-0.943
Br	1.14	23.5	2.96	1139.9	17	-1.197
Rb	2.16	55.9	0.82	403	1	-1.383
Y	1.62	19.8	1.22	600	3	-1.51
Zr	1.45	14.1	1.33	640.1	4	-2.37
Nb	1.34	10.87	1.6	652.1	5	-1.56
Mo	1.3	9.4	2.16	684.3	6	-0.36
Rh	1.25	8.3	2.28	719.675	9	-0.479
Sn	1.41	16.3	1.96	708.6	14	-0.767
Sb	1.4	18.23	2.05	834	15	-0.54
Ba	1.98	39.24	0.89	502.9	2	-1.268
Lu	1.56	17.78	1	523.5	0	-1.65
Hf	1.44	13.6	1.3	658.5	4	-1.59
Ta	1.34	10.9	1.5	761	5	-1.32
W	1.3	9.53	1.7	758.764	6	-0.77
Au	1.34	10.2	2.4	890.1	11	-0.296

Table S3. Comparison of adsorption energy results with first-principle calculations by other scholars.

Atomic species	O	Zr	C	Ta	Ni
This paper	-2.32	-2.37	-1.52	-1.32	-1.50
Pasti[S1]	-2.06	-2.02	-1.46	-1.57	-1.40

[S1] I.A. Pasti, A. Jovanovic, A.S. Dobrota, S.V. Mentus, B. Johansson, N.V. Skorodumova, Atomic adsorption on pristine graphene along the Periodic Table of Elements-From PBE to non-local functionals, Applied Surface Science. 436(1) (2018) 433-440.
